
JOURNAL INFORMATION
==============================
NLM Title Abbreviation: ScientificWorldJournal
Journal ID: ScientificWorldJournal
NLM Journal ID: 101131163
Journal ID: TSWJ

The Scientific World Journal

ISSN: 2356-6140
EISSN: 1537-744X
Publisher: Wiley

ARTICLE INFORMATION
==============================
PMCID: PMC5956299
PMID: 29861679
DOI: 10.1100/tsw.2004.104
Article ID: 979567
Article version: 1
Subjects: Founding Editorial

Founding Editorial

Atala Anthony
Publication date: 2004
Electronic publication date: 2004 Jun 24
Volume: 4
First page: 482
Last page: 482
Copyright: Copyright © 2004 Anthony Atala.
Copyright year: 2004
License: This is an open access article distributed under the Creative Commons Attribution License, which permits unrestricted use, distribution, and reproduction in any medium, provided the original work is properly cited.
License URL: https://creativecommons.org/licenses/by/3.0/

==============================
Founding Editorial
*TheScientificWorldJOURNAL* (2004) 4, 482
ISSN 1537-744X; DOI 10.1100/tsw.2004.104

### Founding Editorial

Anthony Atala
*Editor, Digital Urology Journal*

**E-mail**: aatala@wfubmc.edu

*Received June 9, 2004, 2004; Accepted June 9, 2004; Published June 24, 2004*

**DOMAIN**: urology

When the *Digital Urology Journal* (DUJ) was created and launched in 1996, the term "World Wide Web" was not yet widely in use, but the Editorial Board and I were excited by the potential opportunities of publishing on-line, including a larger and more diverse national and international audience and the ability to publish articles faster than a traditional print journal. At the time, the *Digital Urology Journal* was only the second medical journal to appear on-line and it enjoyed a broad, almost immediate, success with over 1 million hits recorded in just the first month of publication. Now, on-line journals are commonplace and most print journals have an on-line presence. Though thousands of journals are now on-line, only a few retain their "on-line only" status. Eight years later, on-line only journals still have the advantage of faster publication and wider dissemination. The goal of the *Digital Urology Journal* is to continue to broaden its outreach worldwide.

I am delighted that we can now expand our outreach further through our merger with TheScientificWorldJOURNAL. TheScientificWorldJOURNAL has created a novel method of publishing the most current clinical and research articles in a wide variety of disciplines, thereby promoting multidisciplinary communication and exchange. Joining TheScientificWorldJOURNAL as the Urology domain will give our readers a chance to explore other realms of medicine and will greatly benefit our contributors, whose work will be exposed to an even wider audience. In addition, the articles will now be available as references in MedLine, the National Library of Medicine articles database.

All this will be accomplished without affecting the services the *Digital Urology Journal* is proud to provide to our readers, including our on-line calendar of events, which has proven to be a very successful way for conference organizers to reach their target audience: urologic health care providers and researchers.

As one of the few peer-reviewed medical journals that have always been entirely on-line, the *Digital Urology Journal's* new relationship with TheScientificWorldJOURNAL is a natural fit. We are pleased to be part of such a successful and innovative journal and look forward to increased readership and an increased impact, with our articles now indexed by leading services including PubMed/MedLine and permanent archiving by the British Library's Legal Deposit.

**This article should be referenced as follows:**

Atala, A. (2004) Founding editorial. *TheScientificWorldJOURNAL* **4**, 482**.**

©2004 with author.
